# The impact of coronary microvascular dysfunction on the discordance between fractional flow reserve and resting full-cycle ratio in patients with chronic coronary syndromes

**DOI:** 10.3389/fcvm.2022.1003067

**Published:** 2022-10-05

**Authors:** Jacek Legutko, Lukasz Niewiara, Bartlomiej Guzik, Piotr Szolc, Jakub Podolec, Marcin Nosal, Marta Diachyshyn, Krzysztof Zmudka, Paweł Kleczynski

**Affiliations:** ^1^Department of Interventional Cardiology, Institute of Cardiology, Jagiellonian University Medical College, John Paul II Hospital, Krakow, Poland; ^2^Clinical Department of Interventional Cardiology, John Paul II Hospital, Krakow, Poland; ^3^Department of Emergency Medicine, Faculty of Health Sciences, Jagiellonian University Medical College, Krakow, Poland; ^4^Center of Invasive Cardiology, Angiology and Electrotherapy, Krosno, Poland

**Keywords:** coronary microvascular dysfunction (CMD), fractional flow reserve (FFR), resting-full cycle ratio, borderline lesions, coronary artery disease, chronic coronary syndromes, concordance

## Abstract

**Background:**

Resting full-cycle ratio (RFR) is an alternative to fractional flow reserve (FFR) for the evaluation of borderline coronary artery lesions. Although FFR and RFR results are discordant in some cases, factors associated with the discordance remain unclear. The role of coronary microvascular dysfunction (CMD) is discussed as a potential mechanism to explain these discrepancies.

**Aim:**

The study aimed to assess concordance between RFR and FFR in a real-life cohort from a high-volume center regarding the role of CMD.

**Methods:**

Consecutive patients with borderline coronary lesions undergoing coronary functional testing for chronic coronary syndromes were included in the study. Measurements of RFR and FFR were performed alongside additional coronary flow reserve (CFR), resistance reserve ratio (RRR), and an index of microcirculatory resistance (IMR) measurements. CMD was defined according to the current guideline by either IMR ≥25 or CFR ≤2.0 in vessels with no significant stenosis.

**Results:**

Measurements were performed in 157 coronary arteries, in 101 patients, with a median age of 66 y., 74% male, with prior history of arterial hypertension (96%), dyslipidaemia (91%), and diabetes (40%). The median value of vessel diameter stenosis was 45% according to QCA.

Overall, FFR and RFR values were significantly correlated (*r* = 0.66, *p* < 0.001), where positive FFR/negative RFR and negative FFR/positive RFR were observed in 6 (3.8%) and 38 (24.2%) of 157 vessels. The RFR/FFR discrepancy was present in 44 (28%) of measurements. CMD was confirmed in 28 (64%) of vessels with discrepant RFR/FFR and in 46 (41%) of vessels with concordant results (*p* = 0.01). In discordant RFR/FFR vessels, as compared to concordant ones, significantly lower values of CFR [median 1.95 (IQR: 1.37, 2.30) vs. 2.10 (IQR: 1.50, 3.00), *p* = 0.030] and RRR [median 2.50 (IQR: 1.60, 3.10) vs. 2.90 IQR (1.90, 3.90), *p* = 0.048] were observed.

Main predictors of RFR/FFR discrepancy in a univariate regression analysis were: higher age of patients [OR = 1.06 (1.01; 1.10), *p* = 0.010], presence of CMD [OR = 2.51 (1.23; 5.25), *p* = 0.012], lower CFR [OR = 1.64 (1.12; 2.56), *p* = 0.018], and lower RRR values [OR = 1.35 (95% CI: 1.03; 1.83), *p* = 0.038].

**Conclusion:**

In discrepant RFR/FFR vessels, CMD is more prevalent than in concordant RFR/FFR measurements, which can be driven by lower CFR or RRR values. Further research is needed to confirm this observation.

## Introduction

Fractional flow reserve measurement (FFR) is a gold standard to obtain information about ischemia in an invasive setting (1). Nevertheless, full stable hyperaemia is an absolute necessity to get adequate FFR results (2–6).

To avoid this inconvenience, new non-hyperemic invasive indices calculated in different cardiac cycle phases, are being developed and introduced to contemporary practice (7–10). Resting full cycle ratio (RFR) is one of the new non-hyperemic indices, assessed during the whole cardiac cycle, with performance confirmed in real-world practice (9, 11).

Unfortunately, not all measurements of RFR and FFR provide concordant results, and there is a considerable number of discrepancies between those two indices.

Several clinical and angiographic risk factors for this discrepancy have been reported (11–15). A few pathomechanisms of RFR/FFR discrepancy are discussed, however precise data are scarce.

Coronary microvascular dysfunction (CMD) is highly prevalent in patients presenting with chronic coronary syndromes (CCS), and as RFR is a non-hyperemic index, some concerns may arise about the potential role of CMD in a discrepancy between hyperemic FFR assessment and RFR-based decision on revascularization. However, RFR-related data in this context are scarce.

The CMD may be a potential contributor to differences in CFR and RRR values reported in the context of discordance between FFR and another non-hyperemic pressure-derived index, i.e., iFR (16). Similarly, microvascular dysfunction was discussed in terms of RFR and FFR discrepancy, nevertheless, this issue was not directly measured and reported in contemporary literature (15).

## Aim

To assess concordance between RFR and FFR in a real-life cohort from a high-volume center regarding the role of coronary microcirculatory function.

## Materials and methods

The study was a prospective registry of patients with CCS undergoing coronary angiography. All procedures were performed with Helsinki Declaration and were approved by the local bioethics committee. Quantitative coronary angiography (QCA) was performed by an independent core lab analyst blinded to the results of FFR/RFR. Using the guide catheter for calibration and an edge detection system (CAAS 5.7 QCA system, Pie Medical, Maastricht, The Netherlands), the reference vessel diameter and minimum lumen diameter were measured, and the percent diameter stenosis was calculated.

### Physiologic measurements

In all vessels with borderline lesions (i.e., 40–90% of diameter stenosis) both resting (Pd/Pa, resting full-cycle ratio) and hyperemic (FFR) indices were assessed using pressure wire (PressureWire X, Abbott US), with hyperaemia induced by constant infusion of adenosine i.v. according to body weight (140 ug/kg/min) (17, 18). Resting full-cycle ratio was defined as lowered filtered P_d_/P_a_ value during 4 cardiac cycles. Coronary flow reserve and index of myocardial resistance were assessed by room-temperature intracoronary saline infusion and calculated using Coroflow ver. 3 software (Abbott, US). FFR/RFR assessment was performed by an independent analyst, blinded to clinical and angiographic data.

### Cut-off values

Values of FFR ≤0.80 and RFR ≤0.89 were assumed hemodynamically significant, also CFR <2.0 and IMR >25 U were considered abnormal (1).

Coronary microcirculatory dysfunction was defined according to current ESC guidelines as IMR >25U or CFR <2.0 where the lesion was assessed to be hemodynamically non-significant (1).

### Statistical analysis

Continuous data were presented as a mean value with standard deviation for normally distributed variables or by a median with an interquartile range for non-normally distributed values. Categorical data were presented as a percentage of the full group. A comparison of continuous variables was performed using the t-Student test or U-Mann Whitney test according to normality status by the Shapiro-Wilk test. Correlation between continuous values was assessed with Pearson R. Receiver operating curve for RFR to detect FFR <0.80 was analyzed, using Youden criteria to calculate the best RFR threshold.

Logistic regression was used to determine independent RFR/FFR discrepancy predictors, those with *p* < 0.1 in univariate analysis were included in multivariate models. In all analyses, a level of *p* < 0.05 was considered significant.

All analyses were performed in R statistical language (R core group, Vienna, AU), using R-studio ver 1.3, tidyverse packages ecosystem, and ggstatsplot package for graphical presentation of results.

## Results

The analysis included 101 patients with chronic coronary syndromes and a median age of 66 years, of which 26% were women, mostly overweight [median BMI 28.1 kg/m^2^ (IQR 26.0; 31.8)], 44% were current or former smokers, 25 patients had a history of prior myocardial infarction.

The discrepancy between RFR and FFR ischemia assessment in at least one vessel was present in 27 patients (27%).

Most of the patients were treated with ACE inhibitors/ARB and beta-blockers, and 40% had a history of diabetes. Detailed patient characteristics are presented in Table 1.

**Table 1:** Baseline clinical data.

**Characteristic**	***N* = 101^a^**
Age, (years)	66 (59, 73)
**Sex**	
Female	26 (26%)
Male	75 (74%)
BMI, (kg/m^2^)	28.1 (26.0, 31.8)
**Medical history**	
Diabetes	42 (42%)
**Smoking status**	
Never	52 (56%)
Current	19 (20%)
In the past	22 (24%)
Arterial hypertension treatment	97 (96%)
Dyslipidemia treatment	92 (91%)
Prior AMI	25 (24.7%)
**Echocardiography**	
LVEF (%)	55 (50, 60)
LVMI g/m2	108 (89.4; 128)
**Laboratory parameters**	
LDL (mmol/l)	2.22 (1.79, 2.86)
HGB (g/dl)	13.9 (13.1; 15.1)
Serum creatinine (μmol/l)	82.0 (71.0; 93.0)
**Pharmacotherapy**	
ASA	91 (90%)
Beta-blockers	86 (85%)
DHP-Ca clockers	33 (33%)
Non-DHP Ca blockers	9 (9.0%)
ACEI or ARB	91 (91%)
**Patient level RFR/FFR concordance**	
RFR and FFR discordant at least one vessel	27 (27%)
RFR and FFR concordant	74 (73%)

a Median (IQR); *n* (%); ACEI, angiotensin-converting enzyme inhibitors; AMI, acute myocardial infarction; ARB, angiotensin receptor blockers; ASA, acetylsalicylic acid; BMI, body mass index; DHP, dihydropyridine; LDL, low-density lipoprotein; LVEF, left ventricle ejection fraction.

## Per vessel analysis—RFR performance

**Figure 1:**
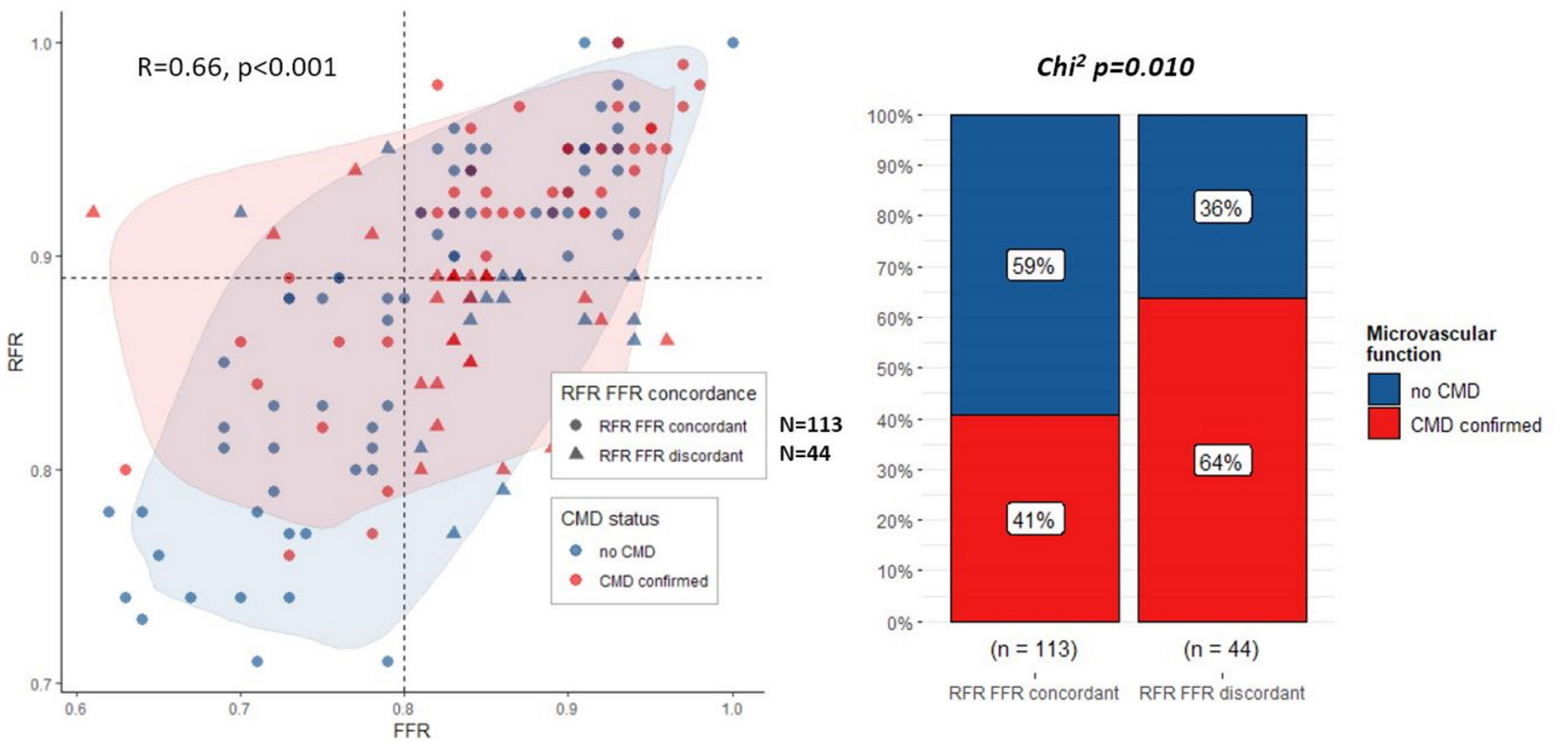
Correlation between RFR and FFR (**left panel**), coronary microvascular dysfunction prevalence (**right panel**).

The analysis included 157 vessels, predominantly left anterior descending arteries (88 vessels), with median artery stenosis of 45% (IQR: 40.50%) and a median FFR of 0.84 (IQR: 0.78, 0.91). Overall, FFR and RFR values showed a good correlation (*R* = 0.66, *p* < 0.001, Figure 1 left panel), while positive FFR with negative RFR and negative FFR with positive RFR were seen in 6 (3.8%) and 38 (24.2%) of 157 vessels, respectively. The discrepancy between RFR and FFR-based decisions on revascularization was present in 44 (28%) of measurements. Discordance was present in 30% of LAD lesions and 26% of non-LAD lesions (*p* = 0.6).

Bland-Altman plot confirmed the moderate agreement of RFR with FFR values, with a median difference between both indices of 0.04 (95% CI 0.02, 0.09, Supplementary Figure 1 Right panel).

AUC for RFR to detect FFR ≤0.80 was 0.865 (95% CI: 0.805–0.925), with an optimal cut-point RFR of 0.88 (see Supplementary Figure 1 Left panel). The diagnostic accuracy of RFR was good, with a sensitivity of 75.9% and specificity of 81.6%.

## Coronary physiology analysis

The presence of CMD was confirmed in 28 (64%) of vessels with discrepant RFR/FFR results and in 46 (41%) of vessels with concordant results (*p* = 0.01, Figure 1 panel right). In discordant RFR/FFR vessels, as compared to concordant ones, significantly lower values of CFR [median 1.95 (IQR: 1.37, 2.30) vs. 2.10 (IQR: 1.50, 3.00), *p* = 0.030] and RRR [median 2.50 (IQR: 1.60, 3.10) vs. 2.90 IQR (1.90, 3.90), *p* = 0.048] were observed. There was no significant difference between discordant and concordant vessels in terms of IMR value [median 22 (IQR: 16, 30) vs. 19 (IQR: 13, 26), *p* = 0.082, respectively]. Detailed results of the angiographic and functional coronary assessment are presented in Table 2.

**Table 2:** Angiographic and functional characteristics of analyzed vessels.

**Characteristic**	**Overall** **(*N* = 157)**	**RFR FFR concordant** **(*N* =113)**	**RFR FFR discordant** **(*N* = 44)**	***P*-value^a^**
**Artery tested**				0.8
LAD	88 (57%)	62 (56%)	26 (60%)	
LCx	39 (25%)	28 (25%)	11 (26%)	
RCA	27 (18%)	21 (19%)	6 (14%)	
**Angiographic analysis**				
QCA DS [%] (IQR)	45 (40, 50)	45 (40, 50)	44 (39, 48)	0.3
Reference diameter [mm] (IQR)	2.7 (2.4; 3.0)	2.7 (2.4; 3.0)	2.6 (2.4; 2.98)	>0.9
Lesion length [mm] (IQR)	17.1 (10.9; 24.7)	17.4 (10.7; 25.0)	16.8 (11.5; 22.5)	0.9
**Epicardial artery stenosis assessment**				
RFR, median (IQR)	0.89 (0.84, 0.94)	0.92 (0.83, 0.95)	0.88 (0.85, 0.89)	**<0.001**
FFR, median (IQR)	0.84 (0.78, 0.91)	0.84 (0.76, 0.91)	0.84 (0.82, 0.86)	0.6
**Coronary microcirculation assessment**				
CMD status, *n* (%)				**0.010**
CMD confirmed	74 (47%)	46 (41%)	28 (64%)	
No CMD	83 (53%)	67 (59%)	16 (36%)	
CFR (IQR)	2.10 (1.50, 2.70)	2.10 (1.50, 3.00)	1.95 (1.37, 2.30)	**0.031**
Tmn resting [s]	0.63 [0.45; 1.01]	0.63 [0.45; 1.00]	0.62 [0.44; 0.97]	0.565
IMR (IQR)	20 (13, 28)	19 (13, 26)	22 (16, 30)	0.082
RRR (IQR)	2.70 (1.80, 3.70)	2.90 (1.90, 3.90)	2.50 (1.60, 3.10)	**0.048**

a Wilcoxon rank sum test; Pearson's Chi-squared test; Fisher's exact test. CMD, coronary microcirculatory dysfunction; CFR, coronary flow reserve; FFR, fractional flow reserve; RFR, resting full-cycle ratio; RRR, relative reserve ratio; QCA, qualitative coronary analysis.

## RFR/FFR discrepancy predictors

Main predictors of RFR/FFR discrepancy in a univariate regression analysis were: higher age of patients [OR = 1.06 (1.01; 1.10) for additional year, *p* = 0.010], presence of CMD [OR = 2.51 (1.23; 5.25), *p* = 0.012], lower CFR [OR = 1.64 (1.12; 2.56) for decrease of 1 unit, *p* = 0.018], and lower RRR values [OR = 1.35 (95% CI: 1.03; 1.83) for decrease of 1 unit, *p* = 0.038].

Lower CFR values, lower RRR values, and the presence of CMD in the analyzed territory, after adjustment for sex and age, remained independent predictors of discordance between RFR and FFR in multivariate regression analysis with OR_adjusted_ = 1.69 (95% CI: 1.15; 2.70, *p* = 0.016), OR_adjusted_ = 1.37 (95% CI: 1.04; 1.89, *p* = 0.024) and OR_adjusted_ = 2.40 (95% CI: 1.15, 5.14, *p* = 0.019), respectively. Detailed results of uni- and multivariate regression analysis are presented in Table 3.

**Table 3:** Univariate and multivariate regression analysis of RFR/FFR discordance predictors.

**Characteristic**	**UnivariateOR (95% CI)**	***P*-value**	**MultivariateOR (95% CI)**	***P*-value**
Age (+ year)	1.06 (1.01, 1.10)	0.009	1.05 (1.01, 1.10)^#^	0.023
Male sex	0.52 (0.24, 1.14)	0.10	0.68 (0.30, 1.55)^##^	0.400
BMI (+1 kg/m^2^)	0.95 (0.86, 1.03)	0.20	—	—
Diabetes	1.36 (0.67, 2.75)	0.40	—	—
**Smoking**		0.19	—	—
Never	Reference		—	—
Current	1.61 (0.60, 4.14)		—	—
In the past	2.19 (0.92, 5.20)		—	—
PAD	0.46 (0.02, 2.96)	0.53	—	—
LVEF (+5% increase)	0.88 (0.73, 1.06)	0.17	—	—
LDL (+1 mmol/l)	0.94 (0.68, 1.26)	0.69	—	—
ACEI or ARB use	0.60 (0.21, 1.87)	0.36	—	—
Beta-blockers use	2.28 (0.81, 8.20)	0.13	—	—
**Vessel tested**		0.75	—	—
LAD	Reference		—	—
LCx	0.94 (0.40, 2.13)		—	—
RCA	0.68(0.23, 1.80)		—	—
**RFR (0.05 lower)**	1.30 (1.01, 1.67)	0.049	1.22 (0.94, 1.61)^###^	0.130
FFR (0.05 lower)	0.93 (0.75,1.14)	0.44	NA	
**CFR (1 unit decrease)**	1.66 (1,13, 2.56)	0.007	1.69 (1.15, 2.70)^###^	0.016
IMR_calc_Yong (1 unit increase)	1.02 (0.99, 1.04)	0.17		
RRR (1 unit decrease)	1.35 (1.04, 1.85)	0.38	1.37 (1.04, 1.89)^###^	0.024
CMD confirmed	2.55 (1.25, 5.33)	0.010	2.40 (1.15, 5.14)^###^	0.019

—Not applicable;

# adjusted for sex only;

## adjusted for age only;

### adjusted for sex and age; ACEI, angiotensin; ARB, angiotensin receptor blockers; BMI, body mass index; CFR- coronary flow reserve; DHP dihydropyridine, CMD, coronary microcirculatory dysfunction; FFR, fractional flow reserve; LAD, left anterior descending, LCx, left circumflex; LDL, low-density lipoprotein; LVEF, left ventricle ejection fraction; PAD, peripheral artery disease; RCA, right coronary artery; RFR, resting full-cycle ratio; RRR, relative reserve ratio.

## Discussion

Resting full-cycle ratio is one of several new, non-hyperemic physiological indices, assessed during a whole cardiac cycle, providing convenient, on-table proof of ischemia.

Several studies showed a significant level of discrepancy between RFR and FFR-based decisions on revascularization (12, 15, 19–21). These studies explored angiographic and clinical markers of this discrepancy. Noteworthy, none of them analyzed the coronary microcirculatory status of patients.

In the current study, we provide additional data validating RFR as a non-hyperemic index in a real-life cohort of patients with chronic coronary syndromes and present evidence for the higher prevalence of coronary microcirculatory dysfunction in patients with discordant RFR and FFR-based decision on revascularization as compared to those with concordant RFR/FFR results.

### RFR performance in intermediate coronary stenosis

Overall, our data confirm a particularly good correlation between RFR and FFR values. A similar, good correlation was described by Svanerud et al. with *R*^2^ = 0.557 (9). Consistently, Ohashi et al. showed an even better RFR to FFR positive correlation (*r* = 0.774, *p* < 0.001) (14). The ICC value showed moderate concordance between RFR and FFR values, however, one needs to remember that RFR, as a non-hyperemic index, records systematically higher values.

An optimal cut-off value of 0.89 to detect significant lesions was originally reported by Svanerud (9), however other authors suggested different values ranging up to 0.90–0.92 (13, 14). In our analysis, the optimal cut-off for RFR was calculated on 0.88, which is similar and concurs with available data.

Regardless of the report, all authors agree there is a considerable level of discrepancy between RFR and FFR-based decisions on revascularization. In our cohort in over one-fourth of measurements, both indices suggested different classifications of lesions. Goto et al. reported a similar level of discrepant measurements, reported in over 19.6% of cases (15). A big-scale retrospective analysis performed by Lee et al. and including 1,024 vessels, suggested a lower number of discrepancies between RFR and FFR measurements, observed in 13.1% of cases.

### Clinical and angiographic risk factors of discrepancy

Reasons for RFR/FFR discrepancies were analyzed by Goto, who suggested, that end-stage renal disease with hemodialysis and the presence of peripheral artery disease were risk factors for low RFR/high FFR phenotype of discrepancy (15). Muroya et al. compared both phenotypes of RFR/FFR discrepancy and reported anemia as a risk factor for high FFR/low RFR phenotype compared to low FFR/high RFR patients (12).

In our analysis, only the higher age of patients remained an independent clinical risk factor for discrepancy.

Currently published data suggest an association between the analyzed vessel and the level of discordance, especially when comparing LAD and non-LAD lesions (14, 15). In our analysis discordance was also numerically more often when LAD lesions were assessed, however, there was no statistically significant difference. Noteworthy, neither percent diameter stenosis, lesion length nor a reference diameter was associated with the discordance, which is consistent with data presented by Goto et al. (15). On the contrary, Wienemann et al. reported focal lesion as a potential risk factor for RFR/FFR discordance (21).

### Coronary microcirculation dysfunction as a potential mechanism of discrepancy

In our study presence of CMD was an independent predictor of RFR/FFR discordance, driven rather by decreased CFR values than elevated coronary microcirculatory resistance.

This is a unique observation regarding RFR validation, as available data focus on clinical and angiographic factors influencing agreement between RFR and FFR assessment (13, 15).

Lower CFR measured by the thermodilution method, as observed in our study in discrepant RFR/FFR cases, can be attributed to both higher baseline flow velocity (meaning the presence of baseline hyperaemia) and decreased ability to accelerate coronary flow (i.e., microvascular dysfunction). Similar reasoning may be referred to low RRR values in discrepant cases. Both mechanisms may be a reason to develop a low RFR/high FFR phenotype of discrepancy.

Our analysis revealed no change in baseline transit time and the observed difference in CFR is probably due to decreased coronary microvascular reactivity. It is particularly important to emphasize a need for resting baseline conditions to perform any functional coronary physiology testing.

On the other hand, high resting index/low FFR discrepancy phenotype may be caused by hyperactivity of coronary microcirculation, a high amount of myocardium supplied by the artery, or a particularly low baseline coronary flow in a specific area (16). In our analysis, neither vessel bed nor increased microvascular reactivity was observed in the discordant RFR/FFR group. Neither of those proposed pathomechanisms was sufficiently researched in terms of RFR/FFR concordance and are only hypotheses to be checked. Further research is needed, as our study was not powered to verify them.

Finally, one should note, that the potential influence of coronary microvascular dysfunction may be less pronounced when the highest-pressure gradient is calculated during the whole cardiac cycle, compared to diastolic-part only calculations, as in the case of iFR.

## Study limitations

Our study has some limitations. Firstly, this is a single-center analysis. Nevertheless, it was performed in a high-volume referral center and included 157 vessels in over 100 patients, showing a real-life population undergoing functional assessment of intermediate coronary lesions.

Secondly, coronary microcirculation was assessed by an invasive thermodilution method. This approach was driven both by pragmatic reasons and by current chronic coronary syndrome guidelines.

Thirdly, the analyzed group consisted only of patients with chronic coronary syndrome. Therefore, obtained results cannot be used in an acute coronary syndrome setting, where coronary microcirculatory dysfunction may be even more prevalent than in a stable group of patients.

Finally, a sparse number of patients in the low FFR/high RFR cohort precluded an in-depth comparison of discrepant phenotypes, which can be improved by extending the study group.

## Conclusion

In discrepant RFR/FFR vessels, CMD is more prevalent than in concordant RFR/FFR arteries. The observed discrepancy may be driven by lower CFR or RRR values rather than elevated IMR levels. Further research on a wider population, in a multi-center setting, is needed to confirm our observation.

## Data availability statement

The raw data supporting the conclusions of this article will be made available by the authors, without undue reservation.

## Ethics statement

The studies involving human participants were reviewed and approved by Jagiellonian University Bioethics Commetee. The patients/participants provided their written informed consent to participate in this study.

## Author contributions

All authors listed have made a substantial, direct, and intellectual contribution to the work and approved it for publication.

## Funding

The research was funded by Jagiellonian University statutory grant (No. K/ZDS/006435).

## Conflict of interest

The authors declare that the research was conducted in the absence of any commercial or financial relationships that could be construed as a potential conflict of interest.

## Publisher's note

All claims expressed in this article are solely those of the authors and do not necessarily represent those of their affiliated organizations, or those of the publisher, the editors and the reviewers. Any product that may be evaluated in this article, or claim that may be made by its manufacturer, is not guaranteed or endorsed by the publisher.

## Supplementary material

Supplementary Figure 1 ROC analysis for RFR to detect FFR ≤0.80 (**left panel**), Bland-Altman plot for RFR-FFR difference (**right panel**).
