# Supplementary figures and images for: The impact of coronary microvascular dysfunction on the discordance between fractional flow reserve and resting full-cycle ratio in patients with chronic coronary syndromes
Source: Front Cardiovasc Med. 2022 Oct 5;9:1003067. doi: 10.3389/fcvm.2022.1003067 (PMC9581189; doi:10.3389/fcvm.2022.1003067)

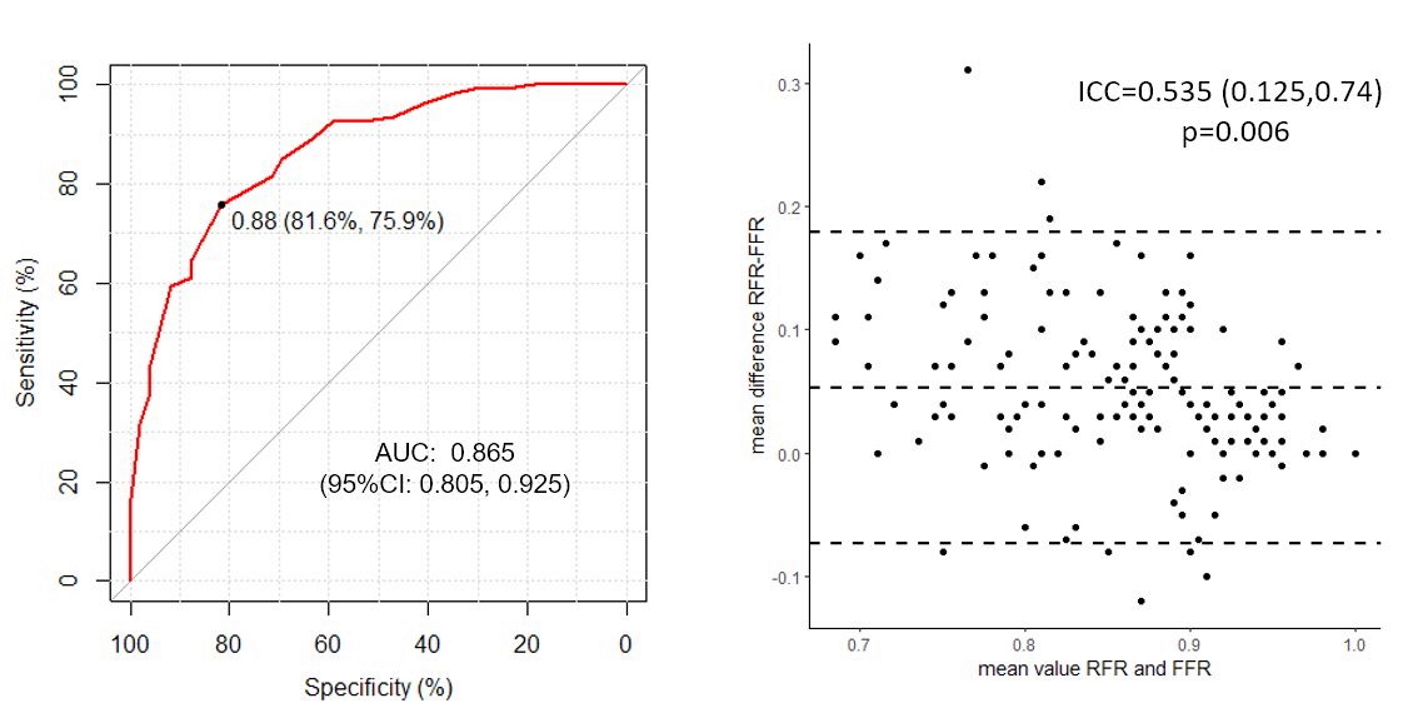

Supplement: Supplementary Figure 1 — ROC analysis for RFR to detect FFR ≤0.80 (left panel), Bland-Altman plot for RFR-FFR difference (right panel). [file Image_1.JPEG]
